# Restriction of Access to Deep Brain Stimulation for Refractory OCD: Failure to Apply the Federal Parity Act

**DOI:** 10.3389/fpsyt.2021.706181

**Published:** 2021-08-12

**Authors:** Rachel A. Davis, James Giordano, D. Brian Hufford, Sameer A. Sheth, Peter Warnke, Alik S. Widge, R. Mark Richardson, Joshua M. Rosenow, Peter Justin Rossi, Eric A. Storch, Helena Winston, JoAnne Zboyan, Darin D. Dougherty, Kelly D. Foote, Wayne K. Goodman, Nicole C. R. McLaughlin, Steven Ojemann, Steven Rasmussen, Aviva Abosch, Michael S. Okun

**Affiliations:** ^1^Department of Psychiatry, University of Colorado Anschutz, Aurora, CO, United States; ^2^Neuroethics Studies Program, Department of Neurology, Pellegrino Center for Clinical Bioethics, Georgetown University Medical Center, Washington, DC, United States; ^3^Zuckerman Spaeder, LLP, New York, NY, United States; ^4^Department of Neurosurgery, Baylor College of Medicine, Houston, TX, United States; ^5^Department of Neurological Surgery, University of Chicago, Chicago, IL, United States; ^6^Department of Psychiatry and Behavioral Sciences, University of Minnesota Medical School, Minneapolis, MN, United States; ^7^Department of Neurosurgery, Massachusetts General Hospital, Boston, MA, United States; ^8^Department of Neurosurgery, Harvard Medical School, Boston, MA, United States; ^9^Department of Neurological Surgery, Northwestern University, Chicago, IL, United States; ^10^University of California San Francisco Department of Psychiatry, San Francisco, CA, United States; ^11^Menninger Department of Psychiatry and Behavioral Sciences, Baylor College of Medicine, Houston, TX, United States; ^12^Denver Health Hospital Authority, Denver, CO, United States; ^13^Springer and Steinberg, PC, Denver, CO, United States; ^14^Department of Psychiatry, Massachusetts General Hospital, Boston, MA, United States; ^15^Departments of Neurosurgery and Neurology, Norman Fixel Institute for Neurological Diseases, University of Florida Health, Gainesville, FL, United States; ^16^Robert J. and Nancy D. Carney Institute for Brain Science, Brown University, Providence, RI, United States; ^17^Butler Hospital, Providence, RI, United States; ^18^The Warren Alpert Medical School of Brown University, Providence, RI, United States; ^19^Department of Neurosurgery, University of Colorado Anschutz, Aurora, CO, United States; ^20^Norman Prince Neurosciences Institute, Rhode Island Hospital, Providence, RI, United States; ^21^Department of Neurosurgery, University of Nebraska Medical Center, Omaha, NE, United States; ^22^Department of Neurology, Norman Fixel Institute for Neurological Diseases, University of Florida Health, Gainesville, FL, United States

**Keywords:** deep brain stimulation, neuromodulation, obsessive-compulsive disorder, OCD, mental health parity, health insurance, advocacy

## Introduction

The Mental Health Parity and Addiction Equity Act of 2008 (MHPAEA) banned the use of differential rules in the provision of mental health care. In an effort to combat discrimination in health insurance against those suffering from mental health conditions, this federal mandate called for large-group employer health insurance plans that cover mental health benefits to provide equivalent coverage for mental health and physical health treatments (1). The parity law did not apply to individual and small-group plans; however, this gap was addressed in the Patient Protection and Affordable Care Act (PPACA or “ACA”). The new act not only required that individual and small-group plans treat behavioral health services with parity, but it also established behavioral health as one of the “essential benefits” required to be offered in the Marketplace plans that were established through the ACA (2). A primary ethical imperative undergirding such legislation is the intersection of the core principles of beneficence, non-malevolence, and justice, wherein moral responsibilities dictate safe and sound clinical intervention (s), which avoid harm, to be equitably provided to those in need. Ethico-legal constructs of non-discrimination and fair access to health care further support these obligations. Yet, despite such ethical and legislative imperatives, there remains a considerable discrepancy between the law and the application of benefits to persons suffering from mental health conditions. It is therefore critical that legal and advocacy groups coordinate their efforts to ensure that behavioral health services for those in need are provided and covered by insurance.

## WIT v. United Behavioral Health

On March 5, 2019, the United States District Court for the Northern District of California in *Wit v. United Behavioral Health* found that United Behavioral Health (“UBH”) breached its fiduciary duties to its insureds under the Employee Retirement Income Security Act (ERISA) by relying upon flawed medical necessity criteria to deny coverage for more than 67,000 claims for treatment of mental and substance use disorders over a period of many years. Specifically, the court found that UBH prioritized its own financial interests above the needs of its insureds by failing to utilize medical necessity criteria that were consistent with generally accepted standards of care (3, 4). While the *Wit* case did not directly assert a parity act violation, which would have required comparing the behavioral guidelines to those used by United Healthcare for medical/surgical services, the *Wit* case's focus on the restrictive coverage policies America's largest insurer used to justify the denial of thousands of behavioral health claims highlights the need for more aggressive oversight of insurers to ensure the provision of appropriate coverage and challenge improper denials of behavioral health claims. This decision may be a watershed moment in the effort to force accountability on insurance companies making coverage decisions relating to behavioral health services. Indeed, former U.S. Representative Patrick Kennedy, the original sponsor of MHPAEA, declared that the “breathtaking” *Wit* ruling was “the Brown v. Board of Education for the mental health movement (5).” Considering this landmark decision, other relevant case law (6), ethical obligations, and our collective experience of repeated restriction of access to appropriate mental health treatment for obsessive-compulsive disorder (OCD), the authors of this opinion article felt compelled to act. We therefore convened a multidisciplinary group of experts in the field of neuromodulation for psychiatric disorders, including neurosurgeons, neurologists, psychiatrists, psychologists, a neuroethicist, and attorneys with expertise in insurance law (including one of the primary attorneys involved in the *Wit* case). Following extensive discussion, we collectively argue for a change in the coverage of mental health services related to psychiatric neuromodulation.

## DBS for OCD and Mental Health Parity Laws

This opinion article is posed as a challenge to the unacceptably common stance of insurers to deny coverage for deep brain stimulation (DBS) for the treatment of severe and intractable OCD (7, 8). Despite the promulgation of generally accepted standards of care for the use of DBS in individuals with severe, disabling, treatment-refractory OCD (9–12), and regulatory approval of DBS via a U.S. Food and Drug Administration (FDA) approved humanitarian device exemption (HDE) (13), insurers commonly refuse to provide reimbursement for this intervention. There are two fundamental problems with the positions that many insurers have taken with regard to this important service. First, they are wrong in concluding that DBS therapy for treatment-resistant OCD should be excluded under applicable health insurance plans as experimental and investigational. In fact, such a position is demonstrably false. The HDE granted by the U.S. FDA (HDE #H050003) on February 19, 2009 (13) provides explicit affirmation that “the probable benefit to health from use of the device outweighs the risk of injury or illness from its use while taking into account the probable risks and benefits of currently available devices or alternative forms of treatment” (14). Investigational devices are covered by Investigational Device Exemptions (IDEs), rather than HDEs (15).

Second, excluding coverage for DBS therapy for OCD is a clear parity violation in light of the fact that major health insurers routinely provide coverage for the use of DBS in the treatment of dystonia, a movement disorder in which a person's muscles contract in a sustained and uncontrollable fashion. Notably, DBS for the treatment of dystonia, which is supported by similar levels of evidence as DBS for OCD (16), is also FDA-approved under an HDE (HDE #020007) (17). The medical policies used by most insurers classify DBS for dystonia, a movement disorder, as medically necessary. For example, since 2005, surgeons at University of Colorado have performed 39 DBS surgeries for dystonia with no denials of coverage. Since 2015, surgeons at University of Colorado have performed 7 DBS surgeries for OCD. Three were covered by Medicare and three by Medicaid. One was denied by private insurance, but the denial was overturned at external review. Coverage for an 8th potential candidate was denied by a private insurer. A 9th patient of the first author had surgery at an outside hospital, and the hospital wrote off charges as charity as well as the family payed out-of-pocket when the claims were denied by a private insurer. Reasons the author have encountered for denial of authorization of DBS for OCD include variations of “experimental and investigational:”

- …investigational and not medically necessary
- …your plan does not cover services that are investigational, meaning there is insufficient evidence to support the efficacy of the treatment compared to standard means of treatment or diagnosis.
- …this service is not considered medically necessary for your condition (obsessive-compulsive disorder) because the plan policy and literature to not support this procedure for your diagnosis as standard of care.
- …your health plan guidelines…show that this type of surgery is unproven because there is not enough evidence that it is effective. It is not medically necessary.
- There are not enough studies to show the device will help your problem. There are not enough studies to show the device is better than other regular treatment for your problem.
- Plan does not cover investigational.
- FDA approval does not obligate us to cover the surgery. We consider it unproven in terms of efficacy.

None of the authors found record of an authorization of DBS for OCD that was denied based on true medical necessity criteria, i.e., the chronicity, refractoriness, and severity of a patient's illness.

There is simply no rational basis to find DBS therapy for OCD to be experimental and investigational, while covering its use for dystonia, given that the status of research and government approval for these two indications is comparable. In fact, the only difference is OCD is a mental health condition, while dystonia is a medical condition affecting motor control. Under the dense terminology of MHPAEA's implementing rules, this appears to be an instance of insurers considering and applying “evidentiary standards” more stringently to mental health benefits than to medical benefits (18). Such practice can—and should—be regarded as discriminatory.

OCD is a debilitating psychiatric condition that occurs in 2.3% of the U.S. population (19). Individuals with OCD experience reduced quality of life, with impairment in multiple domains, and OCD is a chronic condition for most (20–22). Standard-of-care treatment for OCD is exposure and response prevention (ERP), either alone or in combination with serotonergic medication(s). Even with optimal treatment, however, only about 35% of individuals with OCD achieve remission (defined as a Yale-Brown Obsessive Compulsive Scale [Y-BOCS] score of ≤7) (23), and 10–20% remain severely affected (24). DBS is a highly effective treatment option for this group of severe, treatment-refractory patients. Several clinical trials, including some with randomized double-blind sham-controlled designs, have demonstrated positive response rates to DBS (defined as ≥35% YBOCS reduction) in 50–70% of patients (25–29). This success rate is significant given the treatment-refractory nature of these patients. A 2014 systematic review and evidence-based guidelines, conducted and formulated by the American Society for Stereotactic and Functional Neurosurgery (ASSFN) and the Congress of Neurological Surgeons (CNS) (9), as well as a recent update of these guidelines (30), all detail the high level of scientific evidence supporting the use of DBS for treatment-resistant OCD. Treatment guidelines authored by the American Psychiatric Association and the Anxiety and Depression Association of America also recommend the option of DBS for treatment-refractory OCD, if performed at an institution with expertise in both OCD and neuromodulatory surgery (31, 32).

Still, insurers persist in denying coverage for this effective treatment, forcing families, clinicians, and institutions to expend significant time and resources pursuing appeals for authorization of services. Such efforts are rarely successful and frequently cannot even be undertaken, due in large part, to a lack of resources and low resilience among individuals suffering from debilitating illness. A recent review identified private insurance denials as the most frequent factor underlying an appropriate candidate's inability to proceed with DBS surgery (7). Appeal panels and “independent” reviews are often staffed by physicians who lack expertise in neurosurgery or OCD, let alone insight into the use, viability, and value of neuromodulatory surgery for psychiatric disorders. Given the associated costs, the result is that most people who need DBS as the last and best hope for recovery from severely debilitating OCD are unable to obtain it. As one California court noted, an improper denial “following retrospective review will result in the wrongful withholding of payment” while “[a]n erroneous decision in a prospective review” leads to “the withholding of necessary care, potentially leading to a patient's permanent disability or death (33).” This scenario is precisely what occurs when insurers refuse to authorize this medically essential treatment, forcing patients to continue suffering through an otherwise untreatable condition with high potential for long-term disability. Equally alarming are instances in which insurers refuse reimbursement of services rendered, even after they have been pre-authorized (8). This state of affairs imposes ever-increasing burdens on those with mental illness and the healthcare teams caring for them.

## Generally Accepted Standards of Care

The medical necessity criteria for the use of DBS for the treatment of refractory OCD are well-established and accepted by experts in the field of neuromodulation for psychiatric disorders. Medical necessity is based on a determination by the treating psychiatrist's assessment of disability, severity, chronicity, and refractoriness of the illness. These criteria, which have been established based on empirical data, reflect the generally accepted standards of care. We outline these criteria in Table 1 (12, 25, 28, 32, 35–37).

**Table 1:** Medical necessity criteria for the use of DBS for refractory OCD^*^.

Chronicity	OCD Diagnosis for duration of at least 5 years	
Severity	Y-BOCS ≥ 28	
Functional Impairment	In at least one of the domains of: activities of daily living; social functioning; occupational functioning; thinking, concentration, and judgment; or ability to engage in other major life areas (34)	
Treatment-refractoriness	Persistence of severe symptoms (Y-BOCS ≥ 28) despite:	Adequate trials (of at least 12-weeks in duration) of three serotonergic medications at FDA-maximum approved dosages or greater (one of which must be clomipramine) • The requirement for clomipramine may be excluded if the patient experiences adverse effects, does not tolerate the drug, or its use is otherwise contraindicated.
		The use of adjunctive pharmacological agents including a long-acting benzodiazepine and an antipsychotic. • The requirement for a benzodiazepine may be excluded if the patient has a history of substance use disorder or another contraindication to benzodiazepines
		Engagement in exposure therapy for at least 20 sessions with a clinician experienced in the treatment of OCD

* *Generally accepted standards evolve with time, with gains in medical knowledge and development of new treatment options. These criteria should not be considered static or immutable*.

In 2002, to prevent misuses, the OCD-DBS Collaborative Group outlined an ethical framework for performing DBS for OCD (11, 38). The evaluation of individuals and administration of DBS for OCD should be implemented by a multi-disciplinary team, including a stereotactic and functional neurosurgeon, a psychiatrist, an OCD expert therapist, and a neuropsychologist. Individuals must have a thorough psychiatric evaluation, which includes suicide risk assessment, by a psychiatrist who is an expert in the treatment of OCD. Potential candidates for DBS must undergo formal neuropsychological assessment, including cognitive testing, psychiatric interview, evaluation of interpersonal functioning, and discussion of expectations for surgery. Comorbidities must be carefully evaluated and treated before and throughout the process (39, 40). In the United States, institutions must have the requisite IRB approval supporting the FDA HDE.

If insurers are to satisfy generally accepted standards of care, as required by the *Wit* court, they must adopt guidelines that ensure coverage of DBS for OCD when these standards have been met (see Table 1). Their failure to follow such guidelines is inherently improper, just as were UBH's overly restrictive internal guidelines for the provision of behavioral health services.

## Discussion

In conclusion, OCD is associated with a high level of disability, and a small number of individuals have incapacitating illness that is refractory to conventional treatments. We argue that in accordance with current ethico-legal standards, these individuals are eligible for DBS as an option to alleviate their suffering. Extant outcome data support the validity and value of DBS therapy for OCD, and the strength of these data have been recognized both in peer-reviewed guidelines and by the FDA's HDE mark. Thus, we opine that in such cases, the potential benefits of DBS therapy outweigh the risks. The medical policies and practices of insurers who unjustifiably restrict access to DBS in the OCD population are inconsistent with healthcare law. These policies prevent fair access to those individuals most in need, and in denying such coverage, insurers have failed to remain on par with treatment of other medical conditions such as dystonia. We find this position to be both unethical and illegal.

## Endorsement/Support

The International OCD Foundation supports the content of the manuscript titled *Restriction of Access to Deep Brain Stimulation for Refractory OCD: Failure to Apply the Federal Parity Act*. It is critical that individuals suffering from disabling, refractory OCD have access to advanced and effective treatment options, and they should not have to face extra barriers or hurdles simply because the treatment they are seeking is for a mental health condition.

The American Society for Stereotactic and Functional Neurosurgery supports the content of this manuscript, titled *Restriction of Access to Deep Brain Stimulation for Refractory OCD: Failure to Apply the Federal Parity Act*.

## Author Contributions

RD conceived of the manuscript. RD, JG, DH, SS, PW, AW, RR, JR, PR, ES, HW, JZ, DD, KF, WG, NM, SO, SR, AA, and MO contributed directly to the writing and editing of the manuscript. All authors contributed to the article and approved the submitted version.

## Conflict of Interest

RD provides *ad hoc* paid consulting for Medtronic, Inc. She is PI on COMIRB 14-0554: Reclaim® Deep Brain Stimulation Therapy for OCD, and DBS for OCD is ~10% of her clinical practice. RD is a PI (mPI) on the NIH BRAIN RF1 MH121362. She has served on a BrainsWay TMS advisory panel. JG work is supported in part by the Henry M. Jackson Foundation for the Advancement of Military Medicine, Leadership Initiatives, NeurGen, BNB corporation, and the Creighton University Medical Visiting Professorship and receives federal funds from the National Center for Advancing Translational Sciences through the Clinical and Translational Science Awards Program, part of the Roadmap Initiative, Re-Engineering the Clinical Research Enterprise. DH is head of the health insurance litigation practice of Zuckerman Spaeder LLP, where he represents patients and providers in disputes with health insurance companies and claims administrators. He led the team that brought the *Wit v. United Behavioral Health* litigation. SS receives research support from the NIH, McNair Foundation, and Dana Foundation. He is a consultant for Boston Scientific, Zimmer Biomet, Neuropace, Koh Young, and Abbott. PW receives Research Funding though the NIH Brain Initiative and Medtronic. PW is Associate Editor Journal of Neurology, Neurosurgery and Psychiatry. AW has received support from the Minnesota's Discovery, Research, and InnoVation Economy (MnDRIVE) initiative, the Minnesota Medical Discovery Team on Addictions, and NIH; he has received device donations from Medtronic; he has served as a consultant for Circuit Therapeutics, Cyberonics, and Medtronic; and he has multiple patent applications in the area of brain stimulation and circuit modification to improve cognition. RR has received consulting and speaker fees from Medtronic. JR reports grants and personal fees from Boston Scientific. ES receives support from the NIMH RF1 MH121371 grant. WG reports grants from NIH, Biohaven Pharmaceutics, IOCDF, McNair Foundation and non-financial support from Medtronic, personal fees from Biohaven. DD has received honoraria, consultation fees and/or royalties from Medtronic. KF reports grants from NIH, and other funding from Donnellan/Einstein/Merz Chair; grants and non-financial support from Medtronic, grants from St Jude, Functional Neuromodulation, and Boston Scientific, and grants and other funding from Neuropace. Additionally, KF has a patent US 8295935 B2 issued for a DBS cranial lead fixation device. NM receives research funding through NIH (P20GM130452; P50MH106435). MO serves as a consultant for the Parkinson's Foundation, and has received research grants from NIH, Parkinson's Foundation, the Michael J. Fox Foundation, the Parkinson Alliance, Smallwood Foundation, the Bachmann-Strauss Foundation, the Tourette Syndrome Association, and the UF Foundation. MO's DBS research is supported by: NIH R01 NR014852 and R01NS096008. MO is PI of the NIH R25NS108939 Training Grant. MO has received royalties for publications with Demos, Manson, Amazon, Smashwords, Books4Patients, Perseus, Robert Rose, Oxford and Cambridge (movement disorders books). MO is an associate editor for New England Journal of Medicine Journal Watch Neurology. MO has participated in CME and educational activities on movement disorders sponsored by the Academy for Healthcare Learning, PeerView, Prime, QuantiaMD, WebMD/Medscape, Medicus, MedNet, Einstein, MedNet, Henry Stewart, American Academy of Neurology, Movement Disorders Society and by Vanderbilt University. The institution and not MO receives grants from Medtronic, Abbvie, Boston Scientific, Abbott and Allergan and the PI has no financial interest in these grants. MO has participated as a site PI and/or co-I for several NIH, foundation, and industry sponsored trials over the years but has not received honoraria. Research projects at the University of Florida receive device and drug donations. The remaining authors declare that the research was conducted in the absence of any commercial or financial relationships that could be construed as a potential conflict of interest.

## Publisher's Note

All claims expressed in this article are solely those of the authors and do not necessarily represent those of their affiliated organizations, or those of the publisher, the editors and the reviewers. Any product that may be evaluated in this article, or claim that may be made by its manufacturer, is not guaranteed or endorsed by the publisher.
